# Advances in noninvasive diagnosis of renal osteodystrophy

**DOI:** 10.1080/0886022X.2025.2593710

**Published:** 2025-11-27

**Authors:** Qian Yang, Shuang Chen, Zhaolan Yu, Xiangqiong Wen, Santao Ou

**Affiliations:** a Department of Nephrology, The Affiliated Hospital of Southwest Medical University, Luzhou, China; b Sichuan Clinical Research Center for Nephrology, Luzhou, China; c Metabolic Vascular Disease Key Laboratory of Sichuan Province, Luzhou, China

**Keywords:** Renal osteodystrophy, diagnosis, PTH, bone biomarkers, imaging, 18F-NaF PET/CT

## Abstract

Renal osteodystrophy (ROD) refers to the histological abnormalities in bone associated with chronic kidney disease (CKD), closely linked to the progression of CKD, increased risk of fractures, cardiovascular events, and mortality. Early diagnosis and comprehensive assessment of ROD are crucial for enhancing the quality of life of CKD patients and reducing mortality rates. However, the diagnosis and evaluation of ROD remain significant challenges. Although tetracycline double-labeling of iliac crest biopsies is considered the gold standard for diagnosis, its invasiveness and limitations in dynamically monitoring continuous changes in bone morphology restrict its routine application. This article evaluates the research progress in noninvasive diagnostic techniques as alternatives to bone biopsy. These techniques encompass established and emerging bone biomarkers, traditional imaging modalities, and nuclear medicine approaches. The focus is on their application in assessing bone turnover and providing direction for future research. Some individual bone biomarkers and combinations of bone biomarkers have demonstrated considerable potential in the diagnosis of ROD. Quantitative analysis of ^18^F-NaF PET/CT has shown high sensitivity and specificity in diagnosing types of ROD and holds great promise for the evaluation of vascular calcification.

## 1. Introduction

**Figure 1.**
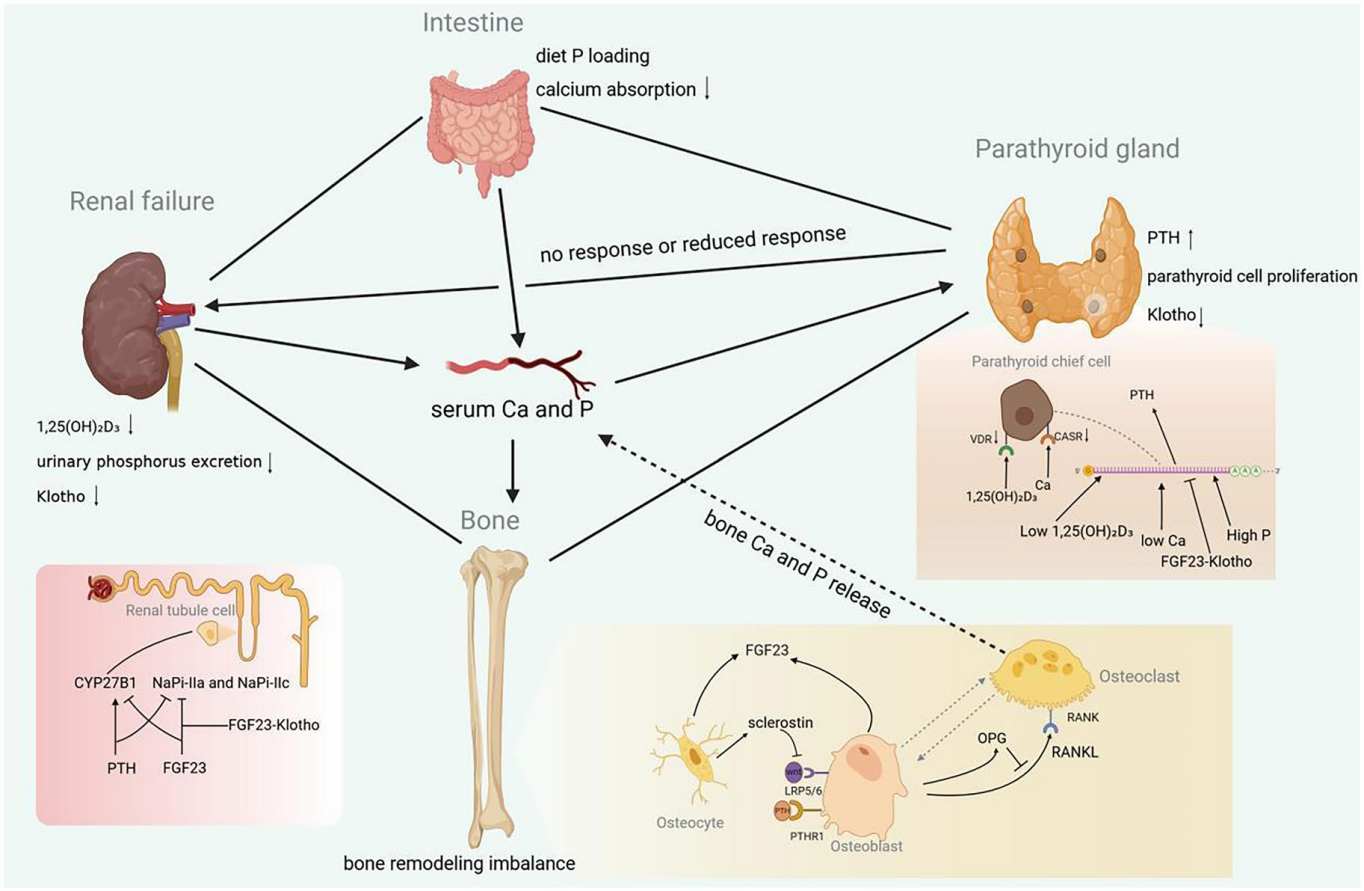
Complex metabolic and hormonal interactions link the kidneys, intestines, parathyroid glands, and skeleton in CKD. As the glomerular filtration rate decreases, the kidney’s ability to excrete phosphorus declines, leading to phosphorus retention and elevated serum phosphorus levels. Initially, osteocytes secrete FGF23, which inhibits renal tubular sodium-phosphate cotransporters (NaPi-IIa/IIc) to boost phosphorus excretion. However, as kidney function deteriorates, the renal response to FGF23 weakens due to reduced Klotho protein, exacerbating phosphorus retention. Renal tubular injury, inflammation, and FGF23′s activation of the FGFR1c-Klotho receptor complex downregulate CYP27B1 (1α-hydroxylase), lowering 1,25(OH)₂D levels [4]. This vitamin D deficiency reduces intestinal calcium and phosphorus absorption, decreasing serum calcium. Yet, with decreased renal phosphorus excretion, serum phosphorus remains high. In SHPT, reduced VDR and CaSR expression, along with low 1,25(OH)₂D and serum calcium, stimulate parathyroid cell proliferation and PTH synthesis [5]. Hyperphosphatemia directly stimulates PTH secretion. These factors worsen SHPT. PTH binds to the PTH1R receptor on osteoblasts, which upregulates RANKL expression, promoting the differentiation of osteoclasts, increasing bone resorption, and contributing to CKD-MBD. Created in https://BioRender.com.

Chronic kidney disease (CKD) has emerged as a significant public health issue, affecting 13.4% of the global population, and is closely associated with various health complications, including cardiovascular, neurological, nutritional, and endocrine disorders [1]. Among these, chronic kidney disease-mineral and bone disorder (CKD-MBD) is a prominent complication. In 2006, the Kidney Disease: Improving Global Outcomes (KDIGO) [2] initiative introduced this term to encompass the following clinical manifestations: (1) abnormalities in the metabolism of calcium, phosphorus, parathyroid hormone (PTH), and vitamin D; (2) Abnormalities in bone turnover, mineralization, volume, linear growth, or strength (i.e., renal osteodystrophy, ROD); and (3) extraskeletal soft tissue and arterial calcification. Given the complexity of bone fragility and overall impairment of bone strength (defined by bone quantity and quality, with the latter including skeletal geometry, microstructure, and tissue properties such as turnover, mineralization, collagen content, and microcracks) in patients with CKD, the 2025 KDIGO guidelines [3] have introduced the new term ‘CKD-associated osteoporosis’. This term integrates ROD and osteoporosis into a unified framework, recognizing and emphasizing that ROD is a skeletal strength disorder that increases fracture risk. The development of ROD is intricately linked to the metabolic and hormonal disturbances induced by CKD (Figure 1), including hyperphosphatemia, hypocalcemia, secondary hyperparathyroidism (SHPT), reduced renal synthesis of 1,25-(OH)_2_D_3_, chronic metabolic acidosis, aluminum toxicity, and chronic inflammation. Subclinical changes in bone metabolism may occur in the early stages of CKD [6], with most patients in stages 3–5 exhibiting skeletal abnormalities and almost all individuals undergoing renal replacement therapy displaying such defects [7,8]. Patients with CKD stages G3–G5D exhibit a 2- to 4-fold higher risk of hip fractures compared to individuals with normal renal function [9,10], with vertebral fractures prevalence reaching 18–55% [11–13]. Abnormal bone remodeling activity is not only associated with an increased risk of fractures but also with increased vascular calcification, including intimal calcification linked to atherosclerotic plaque formation and medial calcification associated with arteriosclerosis, vascular stiffness, and vascular aging [14]. This association increases the risk of cardiovascular events and mortality [15–18]. Therefore, early and precise diagnosis, coupled with timely treatment of ROD, is extremely important. Bone histomorphometric analysis of double tetracycline-labeled iliac bone biopsies is the gold standard for diagnosing ROD [19]. The KDIGO clinical practice guidelines for CKD-MBD recommend the use of bone turnover, mineralization, and volume (TMV) classification (Table 1) to standardize bone biopsy analysis for the comprehensive assessment of tissue pathology [2]. Among these parameters, bone turnover is considered the most critical parameter for managing ROD. Both high and low bone turnover impair the mineral buffering capacity, increasing the risk of fractures and cardiovascular events [20,21]. Additionally, treatment strategies for different types of bone turnover are fundamentally distinct. However, bone biopsy is not routinely evaluated because it is invasive, the biopsy site is confined to the iliac bone, the number of centers that can be evaluated is limited, and it is difficult to monitor over the long term. Therefore, this study systematically reviews advancements in bone biomarkers (including classical indicators and novel molecular markers), imaging techniques, and nuclear medicine methods as alternatives to bone histomorphometry, aiming to achieve early diagnosis, control CKD-MBD progression, guide therapeutic decision-making, and reduce fracture risk.

**Table 1.** TMV classification system for renal osteodystrophy.

Types of ROD	Bone turnover	Bone mineralization	Bone volume^a^
Mild hyperparathyroid-related bone disease	Normal/High	Normal	Low/Normal/High
Osteitis fibrosacystica	High	Normal	Low/Normal
Adynamic bone disease	Low	Normal	Low/Normal
Osteomalacia	Low	Abnormal	Low/Normal
Mixed uremic bone disease	High	Abnormal	Normal

^a^ The bone volume specifically depends on the severity and duration of the disease process.

## 2. Bone biomarkers

Bone remodeling refers to the continuous process in which osteoblasts form new bone while osteoclasts resorb old bone, facilitating self-renewal. It is essential for maintaining bone structure, repairing micro-cracks and fractures, and regulating blood calcium levels. Metabolic byproducts or enzymes generated during this process are classified as bone formation markers and bone resorption markers, collectively known as bone turnover markers (BTMs). Monitoring BTMs and regulators of bone metabolism in blood or urine can rapidly and significantly reflect the overall changes in bone metabolism. These biomarkers are crucial in determining bone turnover types, predicting fracture risk [22–24], monitoring treatment adherence, and evaluating therapeutic efficacy. While they complement histological analysis, they don’t indicate specific bone metabolism sites and aren’t sufficient alone for stratifying fragility fracture risk or monitoring treatment responses [21,25]. Table 2 outlines the roles and mechanisms of these bone biomarkers in bone metabolism.

**Table 2.** The characteristics of bone biomarkers in bone metabolism.

Classification	Biomarker	Characteristics in bone metabolism
Common bone metabolic regulatory markers	PTH	PTH Has a dual effect on bone formation and resorption; continuous high doses of PTH promote bone resorption, while intermittent low doses of PTH promote bone formation [26,27]. PTH binds to PTH1R on target cells, triggering cAMP/PKA and PLC/PKC pathways to regulate blood calcium and bone metabolism [28]. For bone resorption: PTH upregulates RANKL in osteoblasts and downregulates OPG (a decoy receptor for RANKL) via the RANKL-OPG-RANK pathway, enhancing osteoclast activity [29,30]. For bone formation: PTH inhibits Wnt inhibitors in osteoblasts, stabilizing β-catenin complexes and promoting osteoblast differentiation [31]. PTH promotes the differentiation of bone marrow mesenchymal stem cells in the bone marrow into osteoblasts [32].
	Calcitonin	Reduces the number of osteoclasts, inhibits osteoclast activity, and stimulates the proliferation and differentiation of osteoblasts [33]. Inhibits calcium absorption in the small intestine and promotes calcium excretion in urine.
	Vitamin D3	Enhances calcium-binding protein synthesis in the small intestine, increasing calcium absorption and phosphorus uptake. Promotes the reabsorption of calcium and phosphorus in the proximal renal tubules. Interacts with PTH to influence bone formation and resorption. Vitamin D3 can accelerate osteocalcin’s gene and protein expression and enhance osteoblasts’ biomineralization levels [34].
Bone formation markers	ALP; BALP	ALP/BALP hydrolyzes pyrophosphate to relieve mineralization inhibition and generates phosphate to promote calcium and phosphate deposition and bone matrix mineralization. BALP can inactivate and regulate the calcification inhibitor osteopontin through dephosphorylation [35]. BALP is a marker of osteoblast maturity and activity.
	PINP; PICP	Indicates the synthesis of Type I procollagen, reflecting the process of bone formation. PINP is an internationally recognized marker of bone formation [36].
	OC	The Gla residues in OC bind to calcium ions in hydroxyapatite and deposit in the bone matrix to promote mineralization and prevent excessive mineralization [37]. OC regulates mineral maturation and the osteogenic differentiation of mesenchymal stem cells [38]. OC maintains normal bone mineralization, inhibits cartilage mineralization, and prevents abnormal crystal formation [39].
	OPG	Mainly regulates bone metabolism through the OPG/RANK/RANKL system, preventing the binding of RANKL to RANK and inhibiting osteoclast function and promotes bone formation [40]. OPG can bind to and block TNF-related apoptosis-inducing ligand, causing osteoclast apoptosis [41].
Bone resorption markers	TRAP-5b	Mainly originates from osteoclasts. When osteoclasts are active, they release TRAP-5b into the bloodstream during the bone resorption process, reflecting the activity and number of osteoclasts as well as the rate of bone resorption [42].
	CTX; NTX	When osteoclasts resorb the bone matrix, these cross-linked peptides are released into the bloodstream, providing direct information about osteoclasts’ activity and the bone resorption rate. CTX is internationally recognized as a marker of bone resorption [36].
	Pyr; Dpyr	Degradation products of collagen cross-links. Osteoclasts release Pyr and Dpyr when they dissolve the bone matrix during the bone resorption, reflecting bone resorption activity.
Novel bone metabolism markers	Sclerostin	Sclerostin primarily inhibits bone formation by binding to LRP5/6 and Kremen-1 to suppress Wnt/β-catenin signaling [43,44], and also through the LRP4-FGF23/Klotho axis [45]. Sclerostin promotes the formation of osteoclasts and activates osteoclast function in a RANKL-dependent manner, thereby exerting its osteoclastogenic effects [46].
	Dkk1	Dkk1 is expressed by mature osteoblasts and osteocytes, and it regulates bone homeostasis [47]. Dkk1 primarily inhibits the Wnt signaling pathway by binding to LRP6, thereby suppressing the activity and differentiation of osteoblasts, which leads to a reduction in bone formation [48].
	FGF23	FGF23 downregulates the expression of sodium-phosphate cotransporters (such as NaPi-IIa and NaPi-IIc) in the proximal tubules, thereby reducing renal phosphate reabsorption [49]. It suppresses the activity of 1α-hydroxylase, reducing the synthesis of active vitamin D [50]. FGF23 directly inhibited the osteoblastic Wnt pathway through a soluble Klotho/MAPK-mediated process that required Dkk1 induction [51]. Increased bone FGF23 impairs mineralization in CKD-MBD via inhibiting TNAP and causing pyrophosphate buildup [52]. Hypoxia or iron metabolism disorders can upregulate the expression of FGF23 through prolyl hydroxylase Egln1, thereby affecting bone mineralization [53].
	sKlotho	sKlotho inactivates NaPi-IIb in the intestine and NaPi-IIa in the proximal tubules, thereby reducing phosphate absorption and reabsorption [54]. sKlotho activates transient receptor potential vanilloid receptors (TRPV) 5/6, thereby preserving serum calcium and reducing urinary calcium [55,56]. sKlotho interacts with FGFRs in osteoblasts, promoting the FGF23-induced inhibition of cell proliferation and mineralization [57].
	RANKL	Binding to its transmembrane receptor RANK on osteoclast precursors promotes the maturation of osteoclasts, thereby increasing bone resorption and loss.
	Activin A	Activin A is a positive regulator of osteoclast development and bone resorption [58,59]. Activin A can enhance the generation of TRAP-positive osteoclasts [60]. Activin A may act synergistically with RANKL to promote the generation and function of osteoclasts [61]. Activin A activates the Smad2 and MAPK signaling pathways in M-BMMs, including ERK 1/2 and p38, which may be involved in RANKL-induced osteoclastogenesis [62].
	miRNAs	miRNAs play a multifaceted role in bone metabolism, capable of positively or negatively regulating bone formation and bone loss. miRNAs can maintain bone tissue homeostasis through signaling pathways such as the Wnt signaling pathway and the TGF-β/BMP pathway [63].

*Abbreviations*: PTH: Parathyroid hormone; ALP: Alkaline phosphatase; BALP: bone-specific alkaline phosphatase; PINP: Aminoterminal propeptide of type I procollagen; PICP: Carboxy-terminal propeptide of procollagen type I; OC: Osteocalcin; OPG: Osteoprotegerin; TRAP-5b: Tartrate-resistant acid phosphatase 5b; CTX: C-telopeptide of type I collagen; NTX: N-telopeptide of type I collagen; Pyr: Pyridinoline; Dpyr: deoxypyridinolin; Dkk1: Dickkopf1; FGF23: Fibroblast growth factor 23; sKlotho: Soluble Klotho; RANKL: Receptor Activator of NFkappa B ligand; miRNAs: MicroRNAs.

### 2.1. Parathyroid hormone

PTH, an 84-amino acid peptide produced by parathyroid chief cells, critically regulates bone metabolism and calcium-phosphate balance via cross-organ interactions involving bone, intestine, kidney, and parathyroid glands. This balance is disrupted in CKD, leading to SHPT [64]. In severe SHPT, PTH stimulates osteoclast and osteoblast activity, leading to high-turnover bone disease (fibrous osteitis). Conversely, uremia and suppressed PTH cause low bone turnover, commonly referred to as adynamic bone disease (ABD) [65]. PTH levels are linked to fracture and all-cause mortality risks in a ‘U-shaped’ way. Both too high and too low PTH levels can significantly raise these risks [66–68].

PTH is the most widely used noninvasive biomarker for assessing bone turnover in CKD patients. Early studies in the 1980s and 1990s showed a strong correlation between PTH levels and histomorphometric bone parameters [69], but these studies used now-obsolete first-generation assays. Recent studies using second-generation and third-generation immunoradiometric assays indicate that serum PTH levels show weaker correlations with bone turnover compared to other bone biomarkers [20,70,71]. Sprague et al. [20] conducted the largest study on bone histopathology in 492 dialysis patients. They identified optimal intact parathyroid hormone (iPTH) thresholds of 108 pg/mL (low bone turnover) and 323 pg/mL (high bone turnover). However, both thresholds had an area under the receiver operating characteristic curve (AUROC) <0.73, offering no significant advantage over other bone biomarkers. Combining iPTH with bone alkaline phosphatase (BALP) modestly improved predictive capacity. Third-generation assays for whole PTH did not enhance diagnostic accuracy compared to iPTH. Additionally, the KDIGO-recommended PTH target range showed limited diagnostic capability for low bone turnover (65.7% sensitivity, 65.3% specificity) and high bone turnover (37.0% sensitivity, 85.8% specificity). Jorgensen et al. [70] studied 199 CKD stage 4–5D patients and kidney transplant recipients. They found that biointact PTH (whole PTH) (>143.5 pg/mL) had an AUC of 0.77 for diagnosing high bone turnover (70% sensitivity, 74% specificity) and <90.5 pg/mL had an AUC of 0.77 for diagnosing low bone turnover (69% sensitivity, 52% specificity). However, biointact PTH was not superior to other bone biomarkers. The study didn’t assess iPTH, so the benefits of measuring biointact PTH remain unclear.

**Figure 2.**
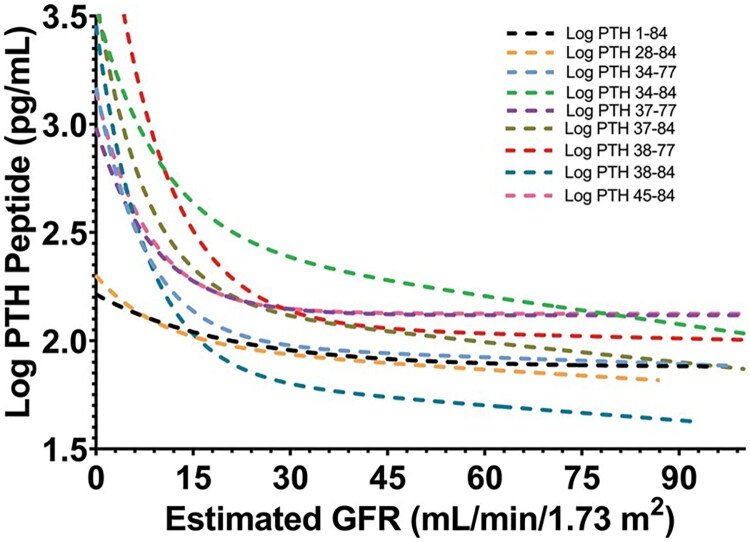
Using liquid chromatography-high resolution mass spectrometry to identify PTH fragments in the circulation of patients with progressive renal failure. Serum concentrations of PTH 1–84 and PTH fragments increased as kidney function declined, and increased significantly when the eGFR decreased to ≤17–23 ml/min per 1.73 m^2^. Reproduced from Kritmetapak, Kittrawee et al. [77] “Chemical Characterization and Quantification of Circulating Intact PTH and PTH Fragments by High-Resolution Mass Spectrometry in Chronic Renal Failure.” Clinical chemistry vol. 67,6 (2021): 843–853, under the terms of the Creative Commons Attribution-NonCommercial-NoDerivs license (CC BY-NC-ND). DOI: https://doi.org/10.1093/clinchem/hvab013.

To improve the predictive value of PTH measurements for bone turnover, prior studies suggested that a ratio of 1–84 PTH to amino-terminally truncated PTH >1 predicts high or normal bone turnover (100% sensitivity), while a ratio <1 indicates low bone turnover (87.5% sensitivity) [72]. Including this ratio in full PTH measurements enhances diagnostic accuracy for bone turnover types [73]. However, other studies found no added value of this ratio in differentiating bone turnover types [74–76]. Our team discovered that the relative levels of iPTH (1–84) and PTH (7–84), rather than their ratio, may be the true determinants of bone turnover. Since PTH (7–84) can counteract high bone turnover, measuring both iPTH and PTH (1–84) together provides a more accurate assessment of bone turnover [76]. Recent research using liquid chromatography–high-resolution mass spectrometry has detected various PTH components in CKD patients’ serum (Figure 2) [77]. Future studies should investigate the bioactivity of these fragments and their correlation with bone tissue morphology to determine if they can more accurately predict bone turnover in ROD patients.

Racial and regional differences in bone turnover types and skeletal responsiveness to PTH have been established in Caucasians versus African Americans [78–81] and in European versus Japanese [82] CKD patients. Thus, each country or region should define optimal PTH ranges for its specific population. A recent small-scale study found iPTH to be the most effective biomarker for detecting high bone turnover in Asian dialysis patients, with an optimal threshold of 484.50 ng/mL (AUC 0.833, sensitivity 0.75, specificity 0.80) [21]. Additionally, different dialysis modalities significantly impact PTH concentrations. A study of peritoneal dialysis (PD) patients showed that 59% exhibited ABD even when iPTH levels met KDIGO guideline recommendations [83]. A retrospective analysis of 491 Chinese PD patients suggested a PTH level of 100–300 pg/mL might be the optimal target for this population [84]. Assessing the appropriate PTH management range for PD patients is crucial.

Overall, while PTH levels typically align with KDIGO guidelines, imbalances in bone turnover, abnormal bone mineral density (BMD), and extensive vascular calcification remain prevalent in dialysis patients [20,21,71]. Undeniably, bone turnover is a progressive process. PTH levels, influenced by hypocalcemia, hyperphosphatemia, and medications, have a short half-life and fluctuate rapidly, failing to accurately reflect histologically observed bone turnover. Moreover, PTH exhibits significant biological variability [85], skeletal resistance to PTH [64], a lack of standardized assays [86], and inherent racial differences. Although PTH extremes provide useful insights into bone turnover, monitoring persistently elevated PTH levels is crucial, as outlined in KDIGO guidelines. However, research on whether PTH trends over time can guide treatment decisions is lacking, underscoring the need for prospective studies. Efforts should also continue to identify more reliable bone biomarkers for enhanced diagnosis.

### 2.2. Bone formation markers

#### 2.2.1. Alkaline phosphatase and bone alkaline phosphatase

Total alkaline phosphatase (tAP), which includes tissue-specific and nonspecific alkaline phosphatases (TNALP), is a common membrane-bound glycoprotein. In serum, bone and liver alkaline phosphatases are the main TNALP subtypes, accounting for over 90% of tAP activity [87]. BALP is primarily produced by osteoblasts, with its principal physiological function being the mineralization of bone matrix and bone formation. BALP correlates more strongly with bone formation rate than tAP [88,89]. Given the impact of liver disease and cross-reactivity with other subtypes, serum BALP is a more specific marker of bone formation. Additionally, its metabolism is independent of renal function and not dialyzable [88], making it a reliable marker for assessing bone turnover in CKD patients. Furthermore, BALP is involved in pathologic calcification associated with vascular calcification [87,90], and elevated serum ALP and BALP levels are linked to increased risks of fractures and cardiovascular events, as well as being an independent predictor of mortality [91–94]. This highlights the importance of managing BALP levels. A recent study suggests that the albumin-to-ALP ratio may be a stronger predictor of mortality than other bone turnover markers [95].

Early clinical studies demonstrated that BALP is significantly correlated with PTH [70,80,88] and multiple dynamic and static bone histomorphometric parameters [80,88,96]. In the diagnosis of low and high bone turnover, the sensitivity and specificity of BALP were found to be comparable to or superior to those of PTH [80,96,97]. Ureña et al. [88] found that plasma BALP levels are higher in maintenance hemodialysis (MHD) patients with high bone turnover than in those with normal or low bone turnover. BALP correlates more strongly with bone formation rate than iPTH [89]. When BALP exceeds 20 ng/mL, either alone or with iPTH >200 pg/mL, it provides optimal sensitivity, specificity, and predictive values for diagnosing high-turnover bone disease. Additionally, BALP outperforms iPTH in diagnosing low-turnover bone disease. Recent studies have also found that BALP is comparable to or superior to PTH in distinguishing bone turnover [20,70,71,98]. Furthermore, BALP is a better biomarker for diagnosing low bone turnover in patients with late-stage CKD and those on dialysis. Jørgensen et al. [70] found that in CKD stages 4–5D and transplant patients, BALP >33.7 μg/L optimally identifies high bone turnover (AUC 0.83), while <24.2 μg/L identifies low turnover (AUC 0.82). These results align with Salam et al. [71], who reported similar cutoffs (>31 μg/L for high turnover, <21 μg/L for low turnover) in similar populations. This suggests cross-population applicability of these cutoffs in advanced CKD, though multicenter validation is needed. The value of BALP in identifying bone turnover in PD patients has also been evaluated. A small-scale, multicenter study determined that a BALP concentration >57.2 U/L is the most specific serum marker for diagnosing high bone turnover in PD patients [99]. Additionally, when liver function is normal, combined assessment of tAP and iPTH provides the most accurate diagnosis of high bone turnover. However, some studies have noted that BALP has a weaker correlation with bone turnover parameters compared to PTH and is less effective than PTH in distinguishing bone turnover [21].

The B1x isoform, one of four BALP isoenzymes, is uniquely found in human bone tissue and the serum of CKD patients. It is most prevalent in dialysis patients (about 60%), occasionally seen in children with CKD [98,100,101]. B1x is associated with lower serum levels of ALP and PTH in CKD patients [98,102]. Haarhaus et al. found in their study of MHD patients that B1x is the only isoenzyme negatively correlated with the number and activity of osteoblasts, and the presence of serum B1x activity is considered an indicator of low bone turnover (AUC 0.83) [98]. However, the value of B1x as a marker for low bone turnover has not been sufficiently explored in further research.

BALP is a promising marker for bone turnover prediction, offering advantages over PTH due to its lower biological and inter-method variability, which enhances its utility in diagnosis and monitoring [97,103,104]. Unlike PTH’s U-shaped curve, BALP’s linear association with mortality may make it a better target for CKD treatment [87]. KDIGO guidelines recommend BALP measurement for assessing bone disease in CKD-MBD patients, but critical levels and treatment targets are yet to be defined. Further studies are needed to confirm its clinical utility and establish treatment thresholds. Despite improvements in commercial immunoassays, challenges in standardization and inter-method variability remain [87,97].

#### 2.2.2. Amino-terminal propeptide of type I procollagen

During bone formation, type I collagen released by osteoblasts is cleaved by specific proteinases into procollagen type I N-terminal propeptide (PINP) and procollagen type I C-terminal propeptide (PICP), which enter the bloodstream and urine, reflecting the rate of bone collagen formation [105]. PINP is a more sensitive marker for assessing bone formation. The International Osteoporosis Foundation (IOF) and the International Federation of Clinical Chemistry and Laboratory Medicine (IFCC) have designated PINP in the blood as the reference marker for bone formation in osteoporosis [36]. PINP exists in two forms in the human body: trimeric and monomeric. The kidneys excrete the monomeric fragment, which accumulates in patients with advanced CKD [106]. Consequently, it is not recommended to use total PINP for assessing bone turnover in late-stage CKD. Using intact PINP, which assesses only the trimeric propeptide, is the most effective way to evaluate bone turnover in ROD patients [106,107]. Previous studies show measuring total PINP is less effective than iPTH and BALP for diagnosing ROD [20], partly for the abovementioned reasons. In a case-control study by Salam et al. [71], total PINP and intact PINP levels were significantly higher in advanced CKD patients, including those on MHD, than in healthy individuals. Intact PINP is more accurate than total PINP for identifying bone turnover types and is comparable to iPTH in distinguishing high-turnover from non-high-turnover bone disease. Additionally, intact PINP outperforms iPTH in identifying low-turnover bone disease, with a threshold of ≤57 μg/L achieving 80% sensitivity, 75% specificity, and an AUC of 0.794. In another study, intact PINP outperformed bioPTH in diagnosing high-turnover and low-turnover bone disease [70]. An intact PINP threshold of 120.7 ng/mL achieved an AUC of 0.85, with 73% sensitivity, 94% specificity, 85% positive predictive value, and 89% negative predictive value for high-turnover bone disease. Combining it with TRAP5b improved diagnostic accuracy to 90%. Intact PINP is promising for managing ROD, but inconsistencies in commercial assays must be resolved first [108]. Global PINP measurement standardization is crucial for international trials, and establishing unified reference ranges and therapeutic goals remains a key objective.

#### 2.2.3. Other bone formation markers

Osteocalcin (OC), a bone matrix protein produced by osteoblasts, serves as a serum marker for bone formation and is thought to regulate bone matrix mineralization. Clinical studies show a significant positive correlation between serum OC levels and bone formation parameters [109–111], with plasma OC levels higher in high bone turnover groups than in normal or low bone turnover groups [110–112]. In a bone biopsy study of 84 patients with advanced CKD, OC levels ≤41 ng/L (<7.0 nmol/L) identified ABD with 83% sensitivity and 67% specificity [96]. Combining it with BALP ≤23 U/L improved specificity to 89%. In another study of 17 hemodialysis patients, OC ≤172 μg/L had an AUC of 0.83 for low bone turnover (83% sensitivity, 86% specificity) and 0.95 for high bone turnover, surpassing PTH [109]. However, some studies indicate that OC’s correlation with bone remodeling parameters does not exceed that of PTH [113,114], and it offers no additional value in distinguishing bone turnover [114,115]. OC carboxylation is regulated by vitamin K, which is often unstable in CKD [116]. Intact OC and its fragments coexist in circulation, and renal impairment affects their concentrations [117].

Osteoprotegerin (OPG) is secreted by osteoblasts and suppresses osteoclastogenesis and bone resorption by inhibiting RANKL-RANK binding. Circulating OPG varies across bone turnover types [118], and combining it with iPTH enhances the ability to distinguish between them [119]. However, few studies have examined its value as a bone turnover marker. OPG levels can also be influenced by multiple factors like inflammation, tumors, and renal function. Recent research has explored OPG as a potential marker for other metabolic complications in CKD, such as anemia, protein-energy wasting, inflammation, and cardiovascular disease [120–122]. A recent German study of 5,217 CKD patients found that serum OPG levels were the most effective biomarker for identifying patients at highest risk of adverse cardiovascular events and mortality [123].

### 2.3. Bone resorption markers

#### 2.3.1. Tartrate-resistant acid phosphatase 5b

TRAP-5b is a dimeric enzyme composed of two identical subunits. It exhibits high activity in acidic environments, hydrolyzes phosphates and pyrophosphates, and promotes bone resorption. TRAP-5b is a sensitive and specific biomarker of osteoclast activity and bone resorption [124,125]. It independently predicts bone loss [126], with elevated levels closely linked to an increased fracture risk [127]. TRAP-5b levels are higher in patients with coronary atherosclerosis than in healthy individuals and correlate with disease severity and extent [128]. It is also an independent predictor of cardiovascular events [129]. Serum TRAP-5b concentration is unaffected by kidney function or dialysis [125,130], making it a promising biomarker for bone metabolism and cardiovascular risk. Previous studies have shown that TRAP-5b links to bone turnover and mineralization parameters [124]. TRAP-5b has high sensitivity and specificity for diagnosing high and low bone turnover [21,70,71]. Additionally, research has shown that TRAP-5b is a better predictor of low bone turnover than PTH [70,71]. Jørgensen et al. [70] showed that TRAP-5b is the most effective biomarker for predicting low bone turnover in CKD stage 4-5D patients and kidney transplant recipients, surpassing Biointact PTH, BALP, and PINP. With a TRAP-5b threshold of <3.44 U/L, it has an AUC of 0.84 for diagnosing low bone turnover, with 73% sensitivity, 74% specificity, and 94% negative predictive value. Its diagnostic capacity for high bone turnover matches Biointact PTH. The combination of TRAP-5b with BALP or PINP enhances diagnostic accuracy for high and low bone turnover. TRAP-5b is an excellent bone turnover marker, but current research data are limited, and the availability of automated assays limits its use.

#### 2.3.2. Other bone resorption markers

Carboxy-terminal telopeptide of type I collagen (CTX), a bone collagen degradation product, is measured as β-isomerized C-telopeptide cross-linked type I collagen (β-CTX) in plasma or serum to reflect bone resorption. The IOF and IFCC have designated β-CTX as a key reference marker for osteoporosis [36], but its role in predicting fracture risk in CKD patients is unclear. β-CTX levels positively correlate with PTH levels [131] and are higher in SHPT patients than in non-SHPT patients in late-stage CKD. Serum CTX can distinguish bone turnover types but has relatively low sensitivity [71]. Moreover, β-CTX levels in the blood are influenced by diurnal variation, glomerular filtration rate, and potential removal during dialysis. Furthermore, the analytical variability of β-CTX is relatively high [132]. Other bone resorption markers, such as amino-terminal telopeptide of type I collagen, pyridinoline, deoxypyridinoline, and hydroxyproline, have limited research supporting their use as alternative markers of bone turnover [115,133], and thus they are not further elaborated upon here.

### 2.4. New markers of bone metabolism

Wnt/β-catenin signaling is essential for nephron formation and renal development, and it also regulates bone formation, remodeling, and ROD progression [134]. Wnt/β-catenin signaling is regulated by several antagonists, with sclerostin and Dickkopf1 (Dkk1) being the most studied. Sclerostin, a glycoprotein encoded by the SOST gene and expressed by mature osteocytes, inhibits canonical Wnt signaling by binding to LRP5/6. It regulates bone metabolism and atherosclerosis, acts as a key negative regulator of bone formation [135,136], and is an independent predictor of bone loss [126]. Research indicates that sclerostin is involved in early CKD-MBD development, with its levels rising before PTH increases due to renal function decline [136,137]. However, whether it increases is due to reduced renal clearance, increased skeletal production, or additional extraskeletal production remains a matter of debate [138]. Most studies show circulating sclerostin is negatively correlated with PTH, bone turnover markers, and bone formation/mineralization parameters [99,136,139,140]. Elevated sclerostin predicts low bone turnover in advanced CKD and dialysis patients [99,141,142]. In a study of 60 MHD patients, Cejka et al. [139] proved sclerostin is an independent predictor of bone turnover and osteoblast numbers, but the ability of sclerostin to make the distinction between low and high bone turnover is actually low. A bone biopsy study of 56 CKD stage 3–4 patients showed that at a sclerostin threshold of 55.5 pmol/L, the AUC for diagnosing low bone turnover was 0.70 (76.5% sensitivity, 64.5% specificity) [142]. Combining Dkk1 improved performance to an AUC of 0.86 (75.0% sensitivity, 85.7% specificity), but prediction of high bone turnover remained limited. Huybrechts et al. [143] found no significant differences in circulating sclerostin across bone turnover categories, and it shows weaker correlations with BTMs (BALP, PINP, TRAP-5b) than cortical bone sclerostin expression. Additionally, whether circulating sclerostin parallels osseous sclerostin levels remains debated. Recent studies show the sclerostin/PTH ratio correlates more closely with bone mineralization and turnover parameters, and has higher diagnostic value for low bone turnover [83,136]. In summary, circulating sclerostin has been proposed for noninvasive ROD diagnosis, but limited research indicates it isn’t superior to biomarkers like iPTH and BALP in diagnosing bone turnover. Variations in detection methods further constrain its clinical applicability [144,145]. The renal excretion of circulating sclerostin requires further investigation, though it is known to be dialyzable [146]. Compared to sclerostin, Dkk1, as an alternative Wnt inhibitor, has been less extensively studied with bone metabolism markers and bone histomorphometric parameters in patients with CKD-MBD [140,147,148]. Furthermore, its potential as a biomarker for bone turnover remains unclear.

The FGF23-Klotho axis inhibits phosphate reabsorption and suppresses 1,25(OH)_2_D synthesis, playing a crucial role in mineral and bone metabolism alterations in CKD [149]. FGF23, a 32 kDa glycoprotein mainly produced by osteocytes and osteoblasts, regulates calcium-phosphate metabolism *via* the bone-kidney-parathyroid feedback loop and influences osteoblast differentiation and matrix mineralization through various mechanisms (Table 2). Studies show that circulating FGF23 levels increase early in renal dysfunction [150,151], preceding noticeable changes in serum phosphate, calcium, or PTH levels [152,153], indicating its early role in CKD-MBD progression. Animal experiments have shown that serum FGF23 levels are significantly correlated with traditional bone biomarkers and histomorphological abnormalities in bone tissue [154]. The use of antibodies against FGF23 can improve SHPT and reverse abnormal bone remodeling activities [155]. Graciolli et al. [141] found that FGF23 negatively correlates with bone formation rate and positively correlates with mineralization lag time across different stages of CKD. FGF23 can be used to identify high bone turnover (AUC 0.779). A recent study showed that while circulating FGF23 alone poorly diagnoses bone turnover, the combination of low FGF23, high α-Klotho, and low PTH has high specificity (100%) but low sensitivity (22%) for identifying low bone turnover in CKD patients [24]. However, FGF23′s value as a bone turnover marker remains uncertain. In a CKD rat model of SHPT, circulating FGF23 levels correlated independently only with bone volume and thickness [156]. Some clinical studies have found that the correlations between FGF23 and bone morphometric parameters contradict theoretical expectations [157,158] and that its ability to distinguish bone turnover is limited [21]. αKlotho is one of the Klotho protein isoforms, expressed primarily in the distal tubules of the kidneys and the parathyroid glands in a membrane-bound form [159], while its soluble form, sKlotho, circulates in the blood, urine, and cerebrospinal fluid [160]. sKlotho regulates calcium-phosphate metabolism and participates in bone remodeling and mineralization through FGF23-dependent and independent mechanisms (Table 2). sKlotho levels decline in CKD patients before serum FGF23 [161,162] and PTH [163,164] increase. Low sKlotho reflects bone microstructural damage, inhibited bone formation, and impaired mineralization in CKD [165,166]. A meta-analysis of 17 CKD studies showed sKlotho is positively correlated with calcium (*r* = 0.14, *I*^2^ = 66%, *p* < 0.05), negatively correlated with serum phosphate (*r* = −0.21, *I*^2^ = 84%, *p* < 0.05), and inversely related to PTH levels (*r* = −0.23, *I^2^* = 40%, *p* < 0.05) [167]. Low sKlotho levels were also significantly associated with increased CKD-MBD risk in CKD patients. The CARTaGENE health study suggests that both the lowest and highest sKlotho levels are associated with fractures in CKD [68]. Yet, few studies have explored its value as a biomarker for bone turnover.

Other novel biomarkers include Activin A, an emerging participant in CKD-MBD. A recent review has elaborated on the mechanisms by which Activin A promotes osteoclastogenesis and bone resorption while inhibiting bone formation, thereby contributing to the pathogenesis of CKD-MBD [168]. Clinical studies have demonstrated that serum Activin A levels begin to rise in stage 2 of CKD [169], and further increase with declining renal function. Moreover, its elevation is significantly associated with all-cause mortality [170]. A cross-sectional study of 104 CKD stage 2–5D patients showed significant differences in serum Activin A levels across different bone turnover [169]. Activin A levels correlated with dynamic bone histomorphometric parameters (e.g., bone formation rate and activation frequency) and effectively predicted high/low bone turnover status (AUC >0.8). In predicting high bone turnover, Activin A had comparable AUC, specificity, and sensitivity to iPTH, ALP, and FGF23. Further research indicated that the circadian rhythm of Activin A is disrupted in CKD patients, suggesting that standardized blood sampling times are needed for accurate clinical interpretation of plasma Activin A levels [171].

**Figure 3.**
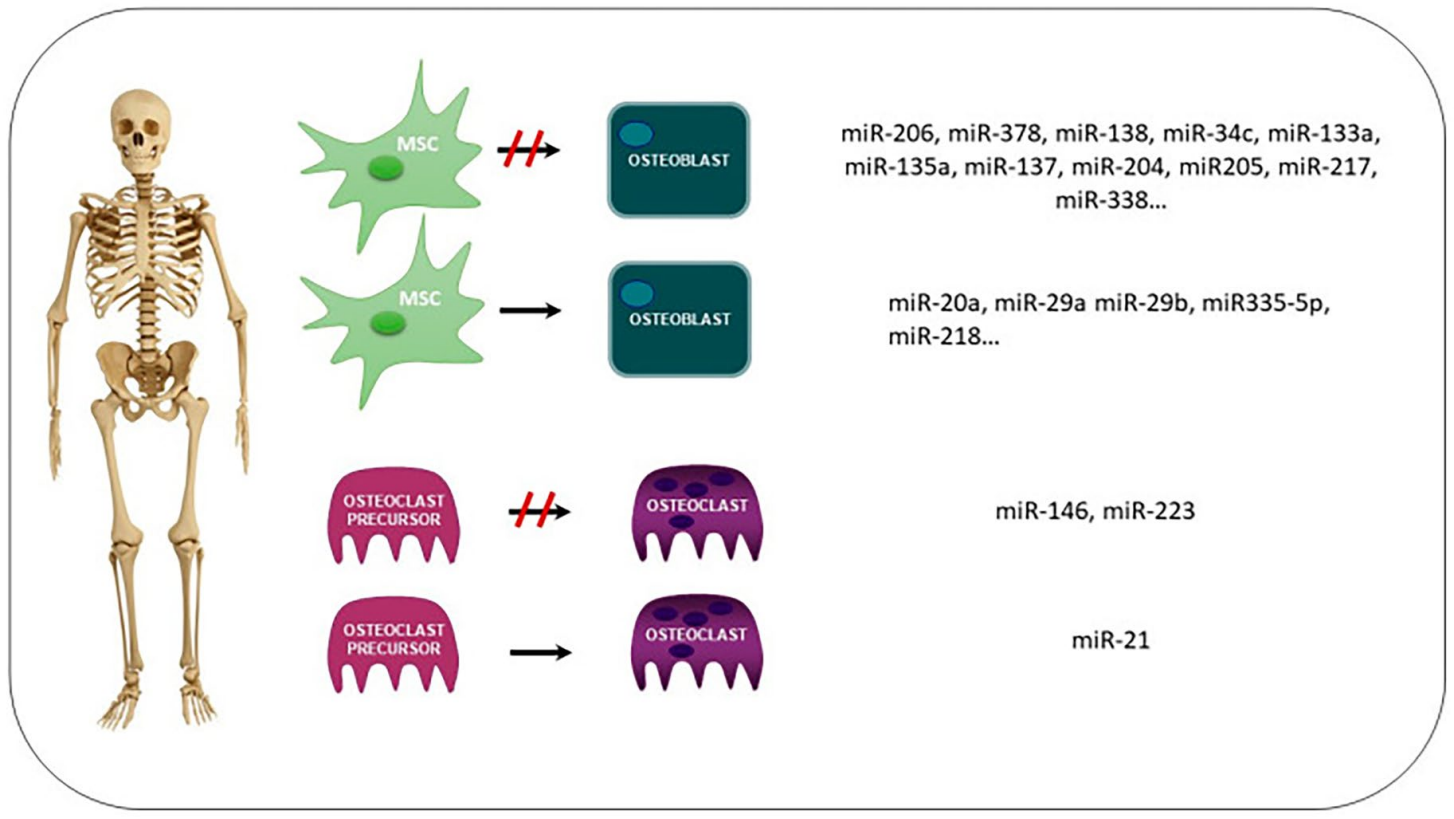
Summary of the role of miRNAs controlling the differentiation and function of osteoblasts and osteoclasts. miRNAs regulate the differentiation of mesenchymal stem cells to osteoblasts and osteoblast differentiation (to inhibit or to promote it). miRNAs can also inhibit or stimulate the differentiation of osteoclasts. Several miRNAs involved in each process are shown in the box (MSC: mesenchymal stem cells). Reproduced from Fernández-Villabrille, Sara et al. [172] “Novel Biomarkers of Bone Metabolism.” Nutrients vol. 16,5 605. 22 Feb. 2024, under the terms of the Creative Commons Attribution License (CC BY). DOI: https://doi.org/10.3390/nu16050605.

MicroRNAs (miRNAs) are key regulators of osteoblast and osteoclast differentiation and function (Figure 3). They are highly stable in blood [173], unaffected by eGFR or dialysis status [174], and thus are promising noninvasive biomarkers. A recent review highlighted their potential as biomarkers in bone diseases, especially osteoporosis [175], but their role in CKD-related bone disease is still unclear. Studies have found that circulating miRNAs may affect PTH secretion. MiR-3680-5p’s target genes can cause PTH and PTH-related protein degradation via the ubiquitin-proteasome pathway [176]. MiR-3680-5p is linked to the lower PTH limit (150 pg/mL) and may serve as a research target for noninvasive biomarkers and ABD etiology. A bone biopsy study of 23 CKD stage 3–5D patients found that miRNA-30b, miRNA-30c, miRNA-125b, and miRNA-155 can differentiate low bone turnover from non-low bone turnover in cortical bone, with AUC values of 0.866, 0.813, 0.813, and 0.723, respectively [177]. When combined, these four miRNAs achieved an AUC value over 0.929, outperforming PTH and BALP used alone or in combination. miRNAs show great potential as biomarkers and novel therapeutic targets for CKD-MBD. However, current data are limited and further research is needed.

## 3. Imaging

### 3.1. Dual-energy X-ray absorptiometry

Dual-energy X-ray absorptiometry (DXA) is a widely employed technique that assesses BMD to evaluate the risk of bone loss and osteoporosis. In CKD, traditional fracture risk factors along with uremic toxins, acidosis, hyperparathyroidism, glucocorticoid use, and the cumulative effects of ROD, collectively impact bone quality and bone strength. A low BMD by DXA may indicate any combination of osteopenia/osteoporosis and superimposed ROD. Undoubtedly, CKD stages G3a–5D patients have higher risks of hip and non-vertebral fractures than the general population, with hip fracture risk rising as glomerular filtration rate declines [10]. Previous studies have demonstrated that low BMD at the peripheral bone and hip predicts fracture risk in CKD [66,178,179]. This prompted the 2017 KDIGO guidelines to recommend BMD measurement in CKD stages 3–5D if the results would influence treatment decisions. A recent prospective study of CKD stages 1–5 patients in the NephroTest cohort showed that after 4.3 years of follow-up, DXA-based longitudinal bone loss mainly occurred at the proximal radius [180]. In a study of 588 dialysis patients, Jaques et al. [181] found that femoral neck BMD independently predicts hip and any-site fractures and mortality. Additionally, low head and pelvis BMD, and low total BMD by whole-body DXA, independently predict increased all-cause and cardiovascular mortality [182]. These findings highlight the potential of cortical-rich sites as key biomarkers for early pathological changes and prognostic indicators in CKD patients, but further research is needed to confirm the clinical significance of different BMD measurement sites. Furthermore, a T-score ≤ −2.5 at the hip or lumbar spine is commonly used as an osteoporosis intervention threshold. However, the 2021 European Consensus Statement indicates that this threshold isn’t fixed for CKD patients and may vary due to country-specific factors, health economics, and individual clinical contexts, potentially requiring higher thresholds (e.g., ≤ −2.0 or ≤ −1.5) [23]. Similarly, diabetic patients have a threshold of ≤ −2.0 [183], but this adjustment for CKD patients needs further validation. However, DXA BMD may underestimate fracture risk in CKD [23,184,185], as it cannot assess bone quality [186] and is influenced by confounding factors such as soft tissue effects, osteoarthritis, degenerative disk changes, vertebral fractures, and aortic calcification, which are common in CKD patients [23,187]. The novelty of 3D-DXA technology lies in its ability to assess cortical and trabecular bone in the proximal femur for bone quality evaluation [188]. Recent studies using 3D-DXA have revealed that cortical parameters in CKD patients deteriorate with worsening kidney function [189]. Moreover, improvements in bone quality parameters (cortical volumetric BMD and surface BMD at the femoral neck) have been observed in kidney transplant recipients after Denosumab treatment, as assessed by 3D-DXA [190]. These findings underscore the broad potential applications of this technology. In terms of identifying bone turnover types, a recent study showed that the Z-score of forearm BMD by DXA was significantly correlated with bone turnover (rho = 0.307; *p* < 0.05), but the Z-score of BMD by DXA did not show significant differences between different bone turnover types, nor could it be used to distinguish the bone turnover status in patients with advanced CKD [71], which is consistent with previous research [21].

Trabecular Bone Score (TBS) is a DXA-derived parameter assessing trabecular microarchitecture *via* gray-scale analysis of lumbar spine images. It provides fracture risk information independent of age, BMD, and other clinical risk factors [191,192]. In CKD patients, TBS correlates with bone parameters at peripheral, vertebral, and hip sites as measured by HR-pQCT and histomorphometry [193–195]. A recent meta-analysis revealed that CKD patients, including those on MHD and kidney transplant recipients, have lower TBS values than controls [196]. Low TBS is linked to damaged bone microarchitecture, older age, low BMD, diabetes, and high BTMs [195,197–199], and may worsen as renal dysfunction progresses [200,201]. However, most studies indicate no significant association between TBS and dialysis duration [191,198,202]. Notably, dialysis and kidney transplant recipients with vertebral or non-vertebral fractures typically display significantly reduced TBS [198,203,204]. Two large retrospective studies [192,201] found that TBS predicts fracture risk in non-dialysis CKD patients, but this association lost significance after multivariable adjustment in Rampersad et al.’s study [201]. Additionally, a cross-sectional study of 41 dialysis patients showed an independent negative correlation between TBS and the 10-year probability of major osteoporotic fractures [205]. However, two prospective cohorts [206,207] found inconsistent associations between TBS and new fractures, possibly due to small sample sizes (*n* = 57 vs. 59) and few fracture events (7 cases each), underscoring the importance of cautious interpretation. Whether TBS independently predicts fracture risk in kidney transplant recipients remains unclear and needs further study [193,208]. To sum up, TBS isn’t conclusively proven as an independent fracture risk predictor for CKD patients, but it’s widely regarded as a useful tool for assessing bone fragility. Combining TBS with BMD and the Fracture Risk Assessment Tool can potentially refine fracture risk assessment [202,209]. Also, TBS might help predict cardiovascular events and mortality in CKD patients [206], though it doesn’t assess bone turnover [194].

### 3.2. Quantitative computed tomography and high-resolution peripheral quantitative computed tomography

Quantitative computed tomography (QCT) provides volumetric BMD for central skeletal sites like the hip and spine, offering advantages over DXA. It also assesses bone microstructure and biomechanical parameters, providing additional insights for evaluating bone strength [210]. Studies show that QCT outperforms DXA in predicting hip bone loss [126,211]. HR-pQCT, a low-dose 3D imaging technique with 82 µm³ resolution, accurately quantifies peripheral bone vBMD, geometric structure, and microarchitectural parameters. Combined with finite element analysis, it also provides additional bone strength parameters. HR-pQCT demonstrates that early-stage CKD is characterized by trabecular bone loss, increased trabecular separation, reduced trabecular vBMD, and cortical thinning [212]. When patients enter the dialysis stage, changes in bone microstructure accelerate, particularly in the weight-bearing tibia [213]. Hemodialysis patients experience more severe cortical and trabecular bone damage compared to those on PD [214]. Moreover, HR-pQCT measurements of bone volume, geometry, and microstructure at the distal radius and tibia can effectively differentiate fracture status [187,215]. However, some studies indicate that the ability of HR-pQCT to predict fracture risk based on bone microstructure does not seem to be superior to that of DXA [216,217].

In assessing bone turnover, HR-pQCT can obtain information about bone turnover status by detecting differences in the bone microarchitecture associated with various types of bone turnover. In SHPT-associated high-turnover bone disease, PTH acts on osteocytes to induce apoptosis and stimulate the secretion of calcification inhibitors [218], resulting in increased cortical porosity and decreased cortical thickness [219]. Fourier transform infrared spectroscopy and nanoindentation analyses reveal that high bone turnover correlates with material and nanomechanical abnormalities, such as a reduced mineral-to-matrix ratio and decreased stiffness. Analyses of bone biopsies demonstrate that low bone turnover is characterized by diminished trabecular bone volume fraction and marked reduction in trabecular thickness [220]. Cortical bone predominates in the hip, radius, and tibia, while trabecular bone predominates in the spine, explaining higher appendicular fracture prevalence in high bone turnover and vertebral fractures in low turnover [221]. Recent studies based on bone histomorphometric parameters have shown that HR-pQCT is helpful in aiding the classification of bone turnover in patients with advanced CKD and those on MHD, particularly radial HR-pQCT parameters for total volumetric bone mineral density and cortical bone volume, which exhibit high diagnostic capacity (AUCs > 0.8) [71,222,223]. However, it is essential to recognize that imaging parameters are static, reflecting the residual effects of accumulated skeletal changes over time, and may not fully capture the current dynamic state of bone turnover. Thus, they cannot serve as an independent surrogate marker for bone turnover.

### 3.3. High-resolution magnetic resonance imaging

High-resolution magnetic resonance imaging (HR-MRI) allows for three-dimensional reconstruction of skeletal morphology and trabecular microarchitecture without the risk of ionizing radiation. To date, only a few studies based on bone biopsies have explored the potential of HR-MRI in assessing cortical and trabecular microarchitectural changes in ROD [224,225]. Sharma et al. [226] demonstrated that MRI-derived measures of trabecular network integrity, such as surface-to-curvature ratio (S/C) and erosion index (EI), are significantly correlated with histologically assessed trabecular bone volume (S/C, *r* = 0.85, *p* = 0.0003; EI, *r* = −0.82, *p* = 0.001), separation (S/C, *r* = −0.58, *p* = 0.039; EI, *r* = 0.79, *p* = 0.0012), and thickness (S/C, *r* = 0.65, *p* = 0.017). Moreover, MRI-derived trabecular parameters show significant correlations with femoral neck BMD. Additionally, various MRI parameters are strongly associated with histomorphometric measurements of mineralization and bone turnover. A single-centre cross-sectional study utilizing MRI revealed significant alterations in bone microarchitecture among SHPT patients who had undergone parathyroidectomy, marked by trabecular deterioration and reduced trabecular and cortical bone volume [227]. Finite element analysis further suggested a decline in skeletal mechanical capacity. However, due to the complexity of the scanning device, it has largely been replaced by HR-pQCT. Recently, micro-MRI also holds considerable value in evaluating trabecular and cortical bone parameters in dialysis patients and kidney transplant recipients [225,228].

## 4. Nuclear medicine imaging techniques: ^18^F-NaF PET/CT

^18^F-sodium fluoride (NaF) PET/CT is a molecular imaging technique that integrates the functional imaging capabilities of PET with the high-resolution anatomical imaging advantages of CT. As a short half-life bone-seeking tracer,^18^F-fluoride has better pharmacokinetics than ^99m^Tc-MDP, with low protein binding, high bone uptake, and rapid plasma clearance [229]. After diffusing from plasma into bone’s extravascular space, fluoride ions preferentially exchange with hydroxyl groups on newly mineralized, active osteoblast surface, forming fluorapatite [230].^18^F-NaF PET/CT clearly shows skeletal morphological changes and evaluates bone blood flow and remodeling [229]. It offers higher resolution and quantitative precision than ^99m^Tc-MDP and is widely used in bone metastasis, osteomyelitis, metabolic bone disease, degenerative bone disorders, and atherosclerotic plaque research [231,232]. Recently, it has drawn interest for assessing systemic and local bone metabolism in CKD patients.

**Figure 4.**
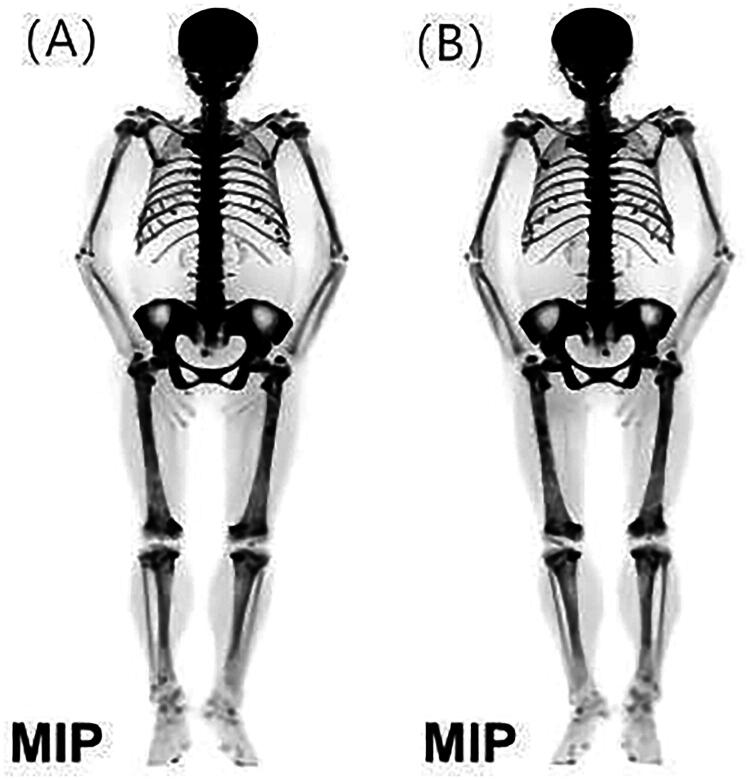
The maximum intensity projection of whole body bone and ^18^F-NaF PET/CT imaging. The images reveal extensive bone density variations and structural irregularities in this patient, with increased ^18^F-NaF uptake, particularly in the skull and mandible (‘black skull sign’) and sternum (‘tie sign’). The distribution is punctate and symmetric, with ‘beaded ribs’ at rib-cartilage junctions. The imaging also presents a ‘super bone scan’ appearance with prominent axial and appendicular skeleton, soft tissue blurring, and no radiotracer excretion in the kidneys or urinary bladder (A: anterior MIP; B: posterior MIP). Reproduced from Xiong, Lin et al. [233] “High turnover renal osteodystrophy due to secondary hyperparathyroidism diagnosed by ^18^F-Fluorocholine combined with ^18^F-NaF PET/CT.” Renal failure vol. 43,1 (2021): 882–885, under the terms of the Creative Commons Attribution License (CC BY). DOI: https://doi.org/10.1080/0886022X.2021.1918165.

**Figure 5.**
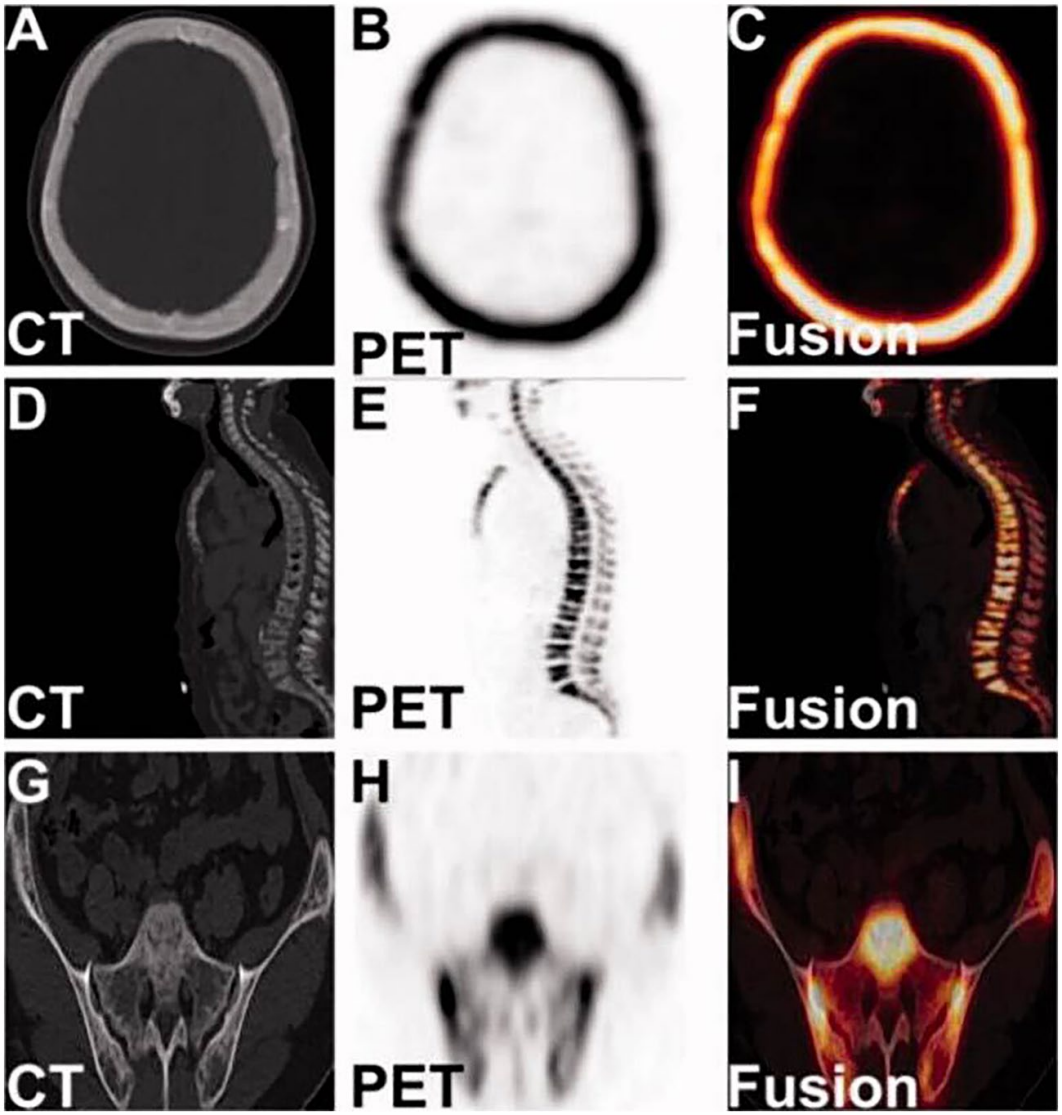
The tomography images of the skull, lumbar spine, and bilateral ilium in ^18^F-NaF PET/CT imaging. Axial CT and PET scans and ^18^F-NaF PET/CT fusion images of the skull, lumbar spine, and bilateral iliac bones show increased density and thickening of the skull’s tables with micronodular osteosclerosis (A–C). Destruction at the edges of several thoracic and lumbar vertebrae with varying degrees of compression from T9 to L1 suggests potential pathological fractures (D–F). Localized bone absorption and destruction on the articular surfaces of the iliac bones, with uneven trabecular density and reduced bone density, indicate possible fibrocystic osteomyelitis (G-I). Reproduced from Xiong, Lin et al. [233] “High turnover renal osteodystrophy due to secondary hyperparathyroidism diagnosed by ^18^F-Fluorocholine combined with ^18^F-NaF PET/CT.” Renal failure vol. 43,1 (2021): 882–885, under the terms of the Creative Commons Attribution License (CC BY). DOI: https://doi.org/10.1080/0886022X.2021.1918165.

^18^F-NaF PET/CT imaging clearly visualizes systemic bone morphology and metabolic status in CKD patients, including localized findings like pseudo-fractures, fibrous dysplasia, and osteosclerosis (Figures 4 and 5) [233,234]. Using bone plasma clearance rate (K_i_) and standardized uptake value (SUV) enable quantitative analysis of bone metabolism and turnover. K_i_ measurements reflect tracer exchange dynamics between plasma and bone mineral, and bone biopsy-based studies show a significant link between regional K_i_ of ^18^F-NaF and bone formation rate [235]. Over the past decade, methods to obtain K_i_ have been explored, including reducing dynamic scan durations to 12–30 min [236,237] and estimating K_i_ via static scans [238–240]. Static scans offer advantages such as ease of use, low invasiveness, multi-region coverage with a single tracer injection, and reduced precision errors. SUV measurement is another semi-quantitative analysis method. Studies show that skeletal SUV measurements correlate moderately to strongly with BALP and PTH in CKD patients [241]. Differences in SUV values were found among bone turnover types, particularly in the L1-L4 vertebrae, bilateral femoral necks, and whole-bone global mean. ROC curve analysis showed excellent AUC values for distinguishing low/normal from high bone turnover, especially in the femur (AUC > 0.87), femoral/hip ratio (AUC 0.84), and GSUV2 (AUC 0.85). However, the study assessed bone turnover using serum PTH levels rather than the gold standard bone biopsy. Additionally, it is important to note that the SUV cannot accurately reflect actual plasma activity because it is influenced by competition from bone lesions in the kidneys and other skeletal areas due to the limited amount of bone tracer given at the time of injection [238]. In patients using drugs like teriparatide, which affects systemic bone metabolism [242], or those with extensive metastatic bone disease [243] or active Paget’s disease [244], SUV changes may partially reflect metabolic changes in other skeletal regions. In these cases, plasma clearance rate may be a more site-specific indicator of bone function than SUV.

To assess the clinical value of this molecular imaging technology in patients with ROD, we reviewed nearly all published English literature on the application of ^18^F-NaF PET/CT or ^18^F-NaF PET as markers for bone turnover in adults with CKD (Table 3). Despite the limited quantity, the results are encouraging. Messa et al. [235] were the first to use ^18^F-fluoride PET to assess bone metabolic activity in ROD patients. They found that the fluoride ion transport rate constant (K) was significantly higher in SHPT patients than in normal subjects and those with low-turnover bone disease. K correlated positively with serum ALP and PTH levels and with histomorphometric indicators of bone formation and erosion. This study shows that ^18^F fluoride PET can distinguish between low-turnover and high-turnover bone diseases and quantitatively estimate osteocytic activity linked to bone histomorphometry. Subsequent studies focusing on patients with MHD have demonstrated that the fluoride bone plasma clearance rate in the regions of interest can effectively identify low and high bone turnover with high sensitivity and specificity, with AUCs exceeding 0.8 [109,246,247]. Moreover, when ^18^F-NaF PET is combined with CT, it not only quantifies bone turnover but also efficiently provides information on bone mass. Aaltonen et al. [247] found that unified classification based on TMV improves the diagnostic accuracy of ^18^F-NaF PET in distinguishing ROD subtypes compared to Malluche’s standard. However, one study showed that the lumbar K_i_ adjusted for BMAD correlates with the mineral apposition rate but not with the bone formation rate [245]. Additionally, no significant differences in K_i_ values were found among dialysis patients, postmenopausal women with osteoporosis, and ABD patients. However, the study’s small sample size and lack of tetracycline-labeled bone biopsies in some patients limit the reliability of the findings for broader populations, highlighting the need for larger-scale studies for further exploration. Additionally, our team evaluated the utility of ^18^F-NaF PET/CT imaging in diagnosing and assessing ROD in MHD patients [248]. We found that 70% of ROD patients exhibited abnormal tracer accumulation at multiple skeletal sites on ^18^F-NaF PET/CT images, consistent with the presentation of superscan. Lumbar spine SUV values were positively correlated with ALP, while femoral neck SUV values showed positive correlations with iPTH, total PINP, and β-CTX. Although our study was not validated by bone biopsy, the findings align with previous research. These studies support the potential of ^18^F-NaF PET/CT imaging as a promising modality for assessing bone metabolism and diagnosing ROD in MHD patients. It sensitively and specifically identifies types of bone turnover, significantly outperforming PTH. When combined with CT, it also quantifies bone quantity and microstructure, identifying high-risk areas for osteoporotic fractures in both cortical and trabecular bone [238]. Numerous studies have also demonstrated that this technology can quantify the direct impact of pharmacological treatment for osteoporosis and other metabolic bone diseases on bone formation in the spine and hip [249,250]. In summary, it is a noninvasive, rapid, and reproducible method for assessing metabolic activity, bone turnover, and microstructure in ROD-affected regions, overcoming BTMs’ limitation of quantifying only overall bone turnover. Moreover, ^18^F-NaF PET/CT can detect microcalcifications [251,252] and high-risk plaques [253], with its quantitative analysis showing a strong correlation with vascular calcification scores and associated biochemical indicators [254,255]. Consequently, it holds significant potential for assessing vascular calcification in patients with CKD. While ^18^F-NaF PET/CT shows promise in diagnosing CKD-MBD, its clinical application in ROD is limited by radiation exposure and high costs.

**Table 3.** studies on ^18^F-NaF PET/CT or ^18^F-NaF PET as markers for bone turnover in Adults with CKD.

Study	Study cohort	Quantitative analysis method	Correlation with bone biomarkers^a^	Correlation to histologic assessment^a^	Diagnostic accuracy
Messa et al. [235] (1993)	11 ROD patients, 11 normal subjects	Three-compartment model nonlinear regression analysis, Patlak analysis.	iPTH: K_PAT_, 0.93; ALP:K_PAT_, 0.81	Osteoid area: K_PAT_, 0.736 Eroded perimeter: K_PAT_, 0.771 Osteoid width: K_PAT_, 0.68 Adjusted apposition rate: K_PAT_, 0.662 Bone formation rate: K_PAT_, 0.835	No data available
Frost et al. [245] (2013)	7 CKD5D patients with suspected ABD, 12 osteoporotic postmenopausal women	Dynamic and single-photon Patlak analysis (for the estimation of Ki in the lumbar spine and other skeletal sites)	No correlation	Model 2 (excluded biopsies with single or no labels): Mineral apposition rate: K_i/BMAD_lumbar spine, 0.81	No data available
Aaltonen et al. [246] (2020)	26 Dialysis patients	Lumbar spine (L1–L4) dynamic scanning: Patlak analysis (net influx rate, K); Pelvic static PET scan: Uptake Fraction Rate (FUR) is an approximation of Patlak K.	PTH: K_i_ mean (L1 – L4), 0.55; FUR mean (iliac crest), 0.58	Bone formation rate per bone surface: K_i_ mean (L1 – L4), 0.63; FUR mean (iliac crest), 0.66 Erosion surface per bone surface: K_i_ mean (L1 – L4), 0.57; FUR mean (iliac crest), 0.60 Osteoclast surface per bone surface: K_i_ mean (L1 – L4), 0.62; FUR mean (iliac crest), 0.62 Osteoblast surface per bone surface: K_i_ mean (L1 – L4), 0.49; FUR mean (iliac crest), 0.58 Mineralized surface per bone surface: K_i_ mean (L1 – L4), 0.55; FUR mean (iliac crest), 0.57 Activation frequency per year: K_i_ mean (L1 – L4), 0.60; FUR mean (iliac crest), 0.65 Osteoid thickness : FUR mean (iliac crest), 0.48	For predict low turnover: ^18^F-Fluoride activity in the PET-scan: Cutoff 0.040 mL/min/mL, AUC = 0.82,76%Sensitivity, 78%Specificity, 64%NPV, 87%PPV PTH: Cutoff < 200 ng/ml, AUC = 0.64, 35%Sensitivity, 78%Specificity, 39%NPV, 75%PPV For predict high turnover: no data available
Vrist et al. [109] (2022)	17 Dialysis patients	The static ^18^F-NaF PET/CT scans were analyzed using the method originally described by Siddique et al. [240]	No data available	Bone formation rate/bone surface: K_i_ (Humeral heads), 0.584; K_i_ (T1–T12), 0.786; K_i_ (L1–L5), 0.780; K_i_ (Pelvis), 0.808; K_i_ (Femoral shafts), 0.748 Activation frequency: K_i_ (Humeral heads), 0.603; K_i_ (T1–T12), 0.766; K_i_ (L1–L5), 0.784; K_i_ (Pelvis), 0.815; Femoral shafts K_i_ (Femoral shafts), 0.694 Osteoid thickness: K_i_ (T1–T12), 0.717; K_i_ (L1–L5), 0.749; K_i_ (Pelvis), 0.612; K_i_ (Femoral shafts), 0.57 Mineralization lag time: K_i_ (Humeral heads), −0.562; K_i_ (T1–T12), −0.626; K_i_ (L1–L5), −0.628; K_i_ (Pelvis), −0.813; K_i_ (Femoral shafts), −0.723 Bone volume per tissue volume :K_i_ (Humeral heads), 0.632; K_i_ (T1–T12), 0.839; K_i_ (L1–L5), 0.802; K_i_ (Pelvis), 0.823; K_i_ (Hip), 0.778	For predict low turnover: When the mean K_i_ (L1-L5) ≤ 0.0295, the AUC is 0.98, with 83% sensitivity, 100% specificity, 100% PPV, and 88% NPV, significantly outperforming bone biomarkers (PTH, OC). For predict high turnover: Mean K_i_ (L1-L5) AUC = 0.90
Aaltonen et al. [247] (2021)	26 Dialysis patients	Lumbar spine (L1–L4) dynamic scanning: Patlak analysis (net influx rate, K); Pelvic static PET scan: Uptake Fraction Rate (FUR) is an approximation of Patlak K.	PTH: K_i_ mean (L1 – L4), 0.63; FUR mean (hip), 0.66	Bone formation rate per bone surface: K_i_ mean (L1 – L4), 0.63; FUR mean (hip), 0.66 Activation frequency per year: K_i_ mean (L1 – L4), 0.63; FUR mean (hip), 0.64 Osteoclast surface per bone surface: K_i_ mean (L1 – L4), 0.66; FUR mean (hip), 0.65 Osteoblast surface per bone surface: K_i_ mean (L1 – L4), 0.52; FUR mean (hip), 0.60 Mineralized surface per bone surface: K_i_ mean (L1 – L4), 0.66; FUR mean (hip), 0.50 Mineral apposition rate:K_i_ mean (L1 – L4), 0.44; FUR mean (hip), 0.53 Osteoid surface per bone surface: K_i_ mean (L1 – L4), 0.66; FUR mean (hip), 0.66 Erosion surface per bone surface: K_i_ mean (L1 – L4), 0.61; FUR mean (hip), 0.60 Osteoid thickness, Ac.f activation fre quency per year: FUR mean (hip) rs = 0.46	For predict high turnover: ^18^F-fluoride activity in the PET scan unified TMV-based (Cutoff >0.055 Ml/min/Ml, AUC = 0.86 82%Sensitivity, 100%Specificity, 88%NPV, 100%PPV), significantly outperforming PTH For predict low turnover: ^18^F-fluoride activity in the PET scan unified TMV-based: Cutoff <0.038Ml/min/Ml, AUC = 0.87 100%Sensitivity 70%Specificity 100%NPV, 50%PPV,significantly outperforming PTH ^18^F-fluoride activity in the PET scan turnover -based: Cutoff <0.038 Ml/min/Ml, AUC = 0.83, 63%Sensitivity, 80%Specificity, 57% NPV, 83%PPV, significantly outperforming PTH
Usmani et al. [241] (2024)	24 ESRD patients, 38 CKD3–4 patients, 10 control	Static PET scanning is used to obtain the SUV uptake values for the entire skeleton and regional bones, including the femoral neck and L1-L4 vertebrae.	BALP: GSUV5, 0.608; GSUV2, 0.535; SUV (R.femur), 0.680; SUV (L.femur), 0.724; SUV (L1), 0.602; SUV (L2), 0.614; SUV (L3), 0.607; SUV (L4), 0.630 iPTH: GSUV5, 0.645; GSUV2, 0.636; SUV (R.femur), 0.698; SUV (L.femur), 0.746; SUV (L1), 0.581; SUV (L2), 0.614; SUV (L3), 0.654; SUV (L4), 0.671	No data available	To discriminate low/normal and high bone turnover: GSUV5, Cutoff =8.31, AUC = 0.735, 38.1%Sensitivity 100%Specificity GSUV2, Cutoff =4.34, AUC = 0.854, 85.7%Sensitivity, 73.3%Specificity SUV (R.femur), Cutoff =5.4, AUC = 0.869, 61.9%, Sensitivity, 100%Specificity SUV (L.femur), Cutoff =5.1, AUC = 0.902, 76.2%, Sensitivity, 100%Specificity SUV (L1), Cutoff =8.73, AUC = 0.783, 71.4%, Sensitivity, 80%Specificity SUV (L2), Cutoff =9.52, AUC = 0.816, 54.8%, Sensitivity, 100%Specificity SUV (L3), Cutoff =8.01, AUC = 0.799, 76.2%, Sensitivity, 73.3%Specificity SUV (L4), Cutoff =8.32, AUC = 0.804, 71.4%, Sensitivity, 80%Specificity

*Abbreviations and definitions*: K_PAT_: A macroparameter estimated via Patlak graphical analysis for ^18^F fluoride ion kinetics: indicating the unidirectional net uptake of the tracer from plasma to bone over 1 h; K_i/BMAD_: K_i_ corrected by BMAD measured via DXA; K_i_ mean (L1–L4) reflects the fluoride activity in the PET scan in the lumbar spine and FUR mean (hip) the fluoride activity at the anterior iliac crest; GSUV2 and GSUV5: Refer to the average SUV obtained when calculating the global mean SUV with threshold values of 2 and 5, respectively.

^a^Values shown are correlation coefficients (*r*).

## 5. Conclusions and perspective

In recent decades, the trend has shifted from high-turnover bone disease due to hyperparathyroidism to ABD [141,256]. The excessive use of vitamin D analogs that inhibit PTH [257], calcium-containing phosphate binders, diabetes, and other factors have increased the prevalence of low bone turnover [258]. Accurately diagnosing the type of ROD is crucial for reducing the risk of fractures, cardiovascular events, and mortality and for determining the appropriate timing of antiresorptive therapy initiation and cessation. Although PTH is still the main biomarker for assessing bone turnover, it is not significantly superior to other bone biomarkers. Bone biomarkers such as BALP, intact PINP, and TRAP-5b exhibit superior potential, and combinations of bone biomarkers have improved diagnostic accuracy for different types of bone turnover. However, most current studies are limited in scale and need standardized detection methods. Moreover, advances in high-resolution imaging help assess ROD patients’ bone microstructure, fracture risk, and turnover status. As a hybrid imaging technique, ^18^F-NaF PET/CT shows great potential in accurately evaluating bone turnover and vascular calcification in CKD-MBD patients, but further validation of its utility in early diagnosis and personalized treatment is needed.
